# Comparison of ^177^Lu-octreotate and ^177^Lu-octreotide for treatment in human neuroblastoma-bearing mice

**DOI:** 10.1016/j.heliyon.2024.e31409

**Published:** 2024-05-18

**Authors:** A. Romiani, K. Simonsson, D. Pettersson, A. Al-Awar, N. Rassol, H. Bakr, D.E. Lind, G. Umapathy, J. Spetz, R.H. Palmer, B. Hallberg, K. Helou, E. Forssell-Aronsson

**Affiliations:** a Department of Medical Radiation Sciences, Institute of Clinical Sciences, Sahlgrenska Academy, University of Gothenburg, Gothenburg, Sweden; b Sahlgrenska Center for Cancer Research, Sahlgrenska Academy, University of Gothenburg, Gothenburg, Sweden; c Medical Physics and Biomedical Engineering, Sahlgrenska University Hospital, Gothenburg, Sweden; d Department of Medical Biochemistry and Cell Biology, Institute of Biomedicine, Sahlgrenska Academy, University of Gothenburg, Gothenburg, Sweden; e Department of Oncology, Institute of Clinical Sciences, Sahlgrenska Academy, University of Gothenburg, Gothenburg, Sweden

**Keywords:** Lutathera, Radionuclide therapy, High-risk neuroblastoma, Somatostatin analogs, Apoptosis

## Abstract

**Background:**

Patients with high-risk neuroblastoma (NB) have a 5-year event-free survival of less than 50 %, and novel and improved treatment options are needed. Radiolabeled somatostatin analogs (SSTAs) could be a treatment option. The aims of this work were to compare the biodistribution and the therapeutic effects of ^177^Lu-octreotate and ^177^Lu-octreotide in mice bearing the human CLB-BAR NB cell line, and to evaluate their regulatory effects on apoptosis-related genes.

**Methods:**

The biodistribution of ^177^Lu-octreotide in mice bearing CLB-BAR tumors was studied at 1, 24, and 168 h after administration, and the absorbed dose was estimated to tumor and normal tissues. Further, animals were administered different amounts of ^177^Lu-octreotate or ^177^Lu-octreotide. Tumor volume was measured over time and compared to a control group given saline. RNA was extracted from tumors, and the expression of 84 selected genes involved in apoptosis was quantified with qPCR.

**Results:**

The activity concentration was generally lower in most tissues for ^177^Lu-octreotide compared to ^177^Lu-octreotate. Mean absorbed dose per administered activity to tumor after injection of 1.5 MBq and 15 MBq was 0.74 and 0.03 Gy/MBq for ^177^Lu-octreotide and 2.9 and 0.45 Gy/MBq for ^177^Lu-octreotate, respectively. ^177^Lu-octreotide treatment resulted in statistically significant differences compared to controls. Fractionated administration led to a higher survival fraction than after a single administration. The pro-apoptotic genes *TNSFS8*, *TNSFS10*, and *TRADD* were regulated after administration with ^177^Lu-octreotate. Treatment with ^177^Lu-octreotide yielded regulation of the pro-apoptotic genes *CASP5* and *TRADD*, and of the anti-apoptotic gene *IL10* as well as the apoptosis-related gene *TNF*.

**Conclusion:**

^177^Lu-octreotide gave somewhat better anti-tumor effects than ^177^Lu-octreotate. The similar effect observed in the treated groups with ^177^Lu-octreotate suggests saturation of the somatostatin receptors. Pronounced anti-tumor effects following fractionated administration merited receptor saturation as an explanation. The gene expression analyses suggest apoptosis activation through the extrinsic pathway for both radiopharmaceuticals.

## 1 Introduction

The role of somatostatin analogs (SSTAs) as tumor-targeting anticancer agents was recognized almost forty years ago. The first somatostatin analogs were shown to positively affect patients with small-intestine neuroendocrine tumors (NETs), reducing symptoms such as diarrhea and flushing following treatment with SSTAs [1]. Furthermore, SSTAs labeled with different radionuclides (e.g., ^68^Ga, ^90^Y, ^111^In, ^161^Tb, and ^177^Lu) have been developed as diagnostic and/or therapeutic alternatives for tumors overexpressing somatostatin receptors (SSTRs). In 1990, a 55-year-old man with metastatic neuroendocrine tumor disease was the first patient treated with a radiolabeled SSTA (^111^In-octreotide) [2]. In 2018 the radiolabeled SSTA ^177^Lu-octreotate was FDA- and EMA-approved for the treatment of patients with SSTR-positive gastroenteropancreatic NETs (GEP-NETs). However, the effectiveness of radiolabeled SSTAs for treating other types of SSTR-expressing tumors is yet to be determined.

Neuroblastoma (NB), categorized as a NET, accounts for 8 % of all childhood cancers and 15 % of cancer deaths in children [[3], [4], [5], [6]]. NB has heterogeneous characteristics, and a diverse set of diagnostic and treatment procedures [5,7]. However, even with increased knowledge regarding the biology of NB and improved treatment modalities, high-risk neuroblastoma (HR-NB) has a 5-year event-free survival (EFS) of less than 50 % [8,9], which emphasizes the need for more effective treatment strategies.

Amplification of *MYCN* is a well-known biomarker for HR-NB, found in approximately 25 % of all NB cases [10]. The oncoprotein MYCN can activate the transcription of genes involved in various cellular processes vital for tumorigenesis, e.g., proliferation, angiogenesis, and survival [10,11]. Despite prior research demonstrating a correlation between *MYCN* expression, tumor relapse and therapy resistance, its complex role is not fully understood yet. Due to the structural similarities of MYCN and other proteins from the MYC family of transcription factors, the specificity of the suggested targeted treatments has been suboptimal [12,13]. Novel treatments seek to find other approaches, e.g., targeting MYCN stability and its cofactors/coregulators, to exploit the amplification of *MYCN* in HR-NB [[14], [15], [16]].

Anaplastic lymphoma kinase (ALK) is another oncoprotein proposed as a target for the therapy of NB. Mutations in the *ALK* gene have been found as the primary oncogenic driver for hereditary NB but are also observed in approximately 8 % of all sporadic cases [17]. The presence of ALK in normal tissue is limited, which is an advantageous premise for targeted treatment [18,19]. *ALK* mutations, most often found in the kinase domain, lead to activation of ALK signaling [20,21]. Lorlatinib, a third-generation ALK TKI, has shown encouraging results regarding anti-tumor effects, overcoming intrinsic resistance observed with ALK kinase domain mutations more effectively than first- and second-generation TKIs (e.g., crizotinib and ceritinib) [[22], [23], [24]]. Despite developing more potent TKIs, drug resistance can develop after long-term treatment [23,25], highlighting the need to understand the underlying biological causes of drug resistance. This also motivates the development of additional targeted treatment types, such as peptide receptor radionuclide therapy (PRRT).

There are five different human subtypes of SSTRs. SSTR2 is the most commonly targeted subtype due to its characteristics and expression in many NETs [26]. In our previous *in-vivo* studies on the NB cell line CLB-BAR, we observed mainly SSTR2 and SSTR3 expression [27]. The expression of the different SSTR subtypes influences the choice of SSTA since the affinity to SSTR subtypes varies much between SSTAs. Reubi et al. observed, for example, that octreotate has a higher affinity to SSTR2 than octreotide, while the opposite is true for SSTR3 [28]. Previous studies have illustrated the different uptake and retention profiles of ^177^Lu-octreotate and ^177^Lu-octreotide and their different therapeutic effects in mice with human small-cell lung cancer and/or the GOT1 NET [29,30]. However, no such comparison has been addressed in NB-bearing mice. Since PRRT with SSTAs has been proposed as an adjunct treatment of SSTR-positive NB, it is of interest to study the influence of the choice of SSTA [[31], [32], [33]].

The aims of the present study were to compare ^177^Lu-octreotate and ^177^Lu-octreotide regarding (1) their uptake and retention in various organs and tissues, and (2) their therapeutic effects in CLB-BAR xenografted mice, as well as (3) their effects on expression of certain apoptosis-related genes in tumor tissue.

## 2 Materials and methods

### 2.1 Radiopharmaceuticals

Both ^177^Lu-octreotate, [^177^Lu-DOTA0, Tyr3]-octreotate (Mallinckrodt Medical BV, NRG, Petten, Netherlands), and ^177^Lu-octreotide, [^177^Lu-DOTA0, Tyr3]-octreotide (Isotopen Technologien München AG, München, Germany) were prepared according to the manufacturer's instructions. The specific activity of ^177^Lu-octreotate and ^177^Lu-octreotide was 25 and 66 MBq/μg, respectively. Radiochemical purity was above 98 % in all studies, determined by instant thin-layer chromatography Silica-Gel (ITLC-SG, chromatography paper 50/PK, Varian, USA) with 0.1 M sodium citrate as the mobile phase. The activity in each syringe was measured using an ionization chamber (CRC-15, Capintec, IA, USA) before and after administration.

### 2.2 Animal model

The study was performed using the HR-NB cell line CLB-BAR with *MYCN/ALK* amplification [34,35], and the cells were cultivated as previously described [27]. Five to six weeks old female BALB/c nude mice (Janvier Labs, France, and Charles River Laboratories, Inc, UK) were injected subcutaneously in one flank with 2 × 10^6^ CLB-BAR cells. Approximately 4–6 weeks after the transplantation, most of the mice had developed tumors (volume range: 200–400 mm^3^) and were included in the study. All animal experiments were approved by the Swedish Ethical Committee on Animal Experiments in Gothenburg (ethical reference numbers: 107-15 and 2779-20) and carried out following guidelines from Animal Research: Reporting of In Vivo Experiments (ARRIVE).

### 2.3 Biodistribution study

For the biodistribution study, mice were administered with either 1.5 or 15 MBq of ^177^Lu-octreotide, and euthanized after 1, 24, or 168 h (n = 5–6/group). The euthanasia procedure was carried out under anesthesia by intraperitoneal injection with pentobarbitalnatrium (60 mg/ml, Apotek Produktion & Laboratories AB, Sweden), followed by cardiac puncture. Thereafter, adrenal glands, blood, bone marrow (collected by scraping the inner part of the femur with a needle), brain, kidneys, liver, lungs, spleen, and tumor were collected and weighed. As previously described [27], the ^177^Lu activity in each sample was measured with a Wallac 1480 NaI (Tl) gamma counter (Wallac Oy, Turku, Finland), and activity concentration levels were determined as percent of injected activity per mass of tissue (%IA/g). Subsequently, tumor-to-normal-tissue activity concentration ratios (T/N) were determined. The biodistribution data was compared with previously published corresponding data for ^177^Lu-octreotate [27].

### 2.4 Therapy studies

Two different therapy studies were designed, one in which the activity levels were increased and one in which the administration of ^177^Lu-octreotate was fractionated. In the former, mice were intravenously (i.v.) injected with 15, 30, or 60 MBq ^177^Lu-octreotate or ^177^Lu-octreotide (n = 4–5/group). In the fractionation study, mice were injected with 1 × 15 MBq, 2× 7.5 MBq with a 2h interval, or 3× 5 MBq with a 1h interval with ^177^Lu-octreotate. A control group (mice received saline i.v. of same volume as the drugs) was used for comparison to the animal groups in both therapy studies. The tumor volume for each animal was measured with a digital caliper twice a week before and after administration. Tumor volume was estimated based on the shape of an ellipsoid and calculated from the measured length, width, and height. Mice were euthanized using the same procedure described above for the biodistribution study, when tumor volume reached 10 % of the body weight, or in case they demonstrated signs of poor health. Tumor samples were immediately frozen in liquid nitrogen and stored at −80 ^ͦ^C for further analysis.

### 2.5 ^177^Lu dosimetry

The mean absorbed dose, D, was determined based on the equation from Medical Internal Radiation Dose (MIRD) Pamphlet No. 21 [36].

$$D\left(r_{T},T_{D}\right)=\frac{\tilde{A}\left(r_{S},T_{D}\right)\sum_{i}E_{i}Y_{i}\emptyset \left(r_{T}\leftarrow r_{s},E_{i},T_{D}\right)}{M\left(r_{T},T_{D}\right)}\text{.}$$

The mean absorbed dose for each tissue was determined as previously described [27]. The time-integrated activity, $\tilde{A,}$ was estimated based on data from the biodistribution study with ^177^Lu-octreotide, where an exponential function was fitted to the activity concentration data for each tissue, and integration was made to eternity. The source tissue and the target tissue were assumed to be the same. Only contribution from electrons were considered. The absorbed fraction, Ø, was set to 1. The mean absorbed dose for each tissue was compared with corresponding data from our previous biodistribution studies of ^177^Lu-octreotate in the same animal model [27].

### 2.6 RNA extraction and RT^2^-PCR analyses

RNA was extracted from tumor samples using RNeasy Lipid Tissue Mini Kit (QIAGEN, Valencia, USA) following the manufacturer's protocol. Briefly, ≤100 mg of a frozen tumor biopsy was added to Qiazol and lysed using Tissue Lyser LT for 6 min. Then, 200 μl chloroform was added and vortexed vigorously, and each sample was incubated at room temperature for 3 min. After centrifugation, the aqueous phase (450 μl) was transferred to RNeasy membranes together with 450 μl 70 % ethanol. After multiple washing steps, 40 μl RNase‐free water was added to the center of the membranes, and total RNA was eluted by centrifugation. The RNA purity, integrity, and concentration were determined by Nanodrop 1000 Spectrometer (Thermo Scientific), 4200 TapeStation (Agilent Technologies) (RIN >8), and Qubit 3.0 Fluorometer (Thermo Fisher Scientific), respectively.

To generate cDNA, the QIAGEN RT2 First Strand Kit (QIAGEN, Valencia, USA) was used. Prepared cDNAs were then mixed with RT^2^ SYBR Green Mastermix (QIAGEN, Valencia, USA), and analyzed in an RT^2^ profiler polymerase chain reaction (PCR) array 96-well plate containing primers with specific targets for 84 genes involved in apoptosis (PAHS-012Z, QIAGEN, Valencia, USA). The other 12 wells contained five housekeeping genes (*ACTB*, *B2M*, *GAPDH*, *HPRT1*, and *RPRP0*) for data normalization and control elements for the determination of genomic DNA contamination and reverse transcription control. The gene expression was assessed using a 7500 Fast Real-Time PCR System (Applied Biosystem™). The fold change (FC) value was obtained using the 2^–ΔΔCt^ method [37]. A │FC│ cutoff of ≥1.5 was applied.

### 2.7 Statistical analyses

Data analyses was performed in GraphPad Prism 9 and Excel (Version 2210). Results from tumor volume measurements and the biodistribution were expressed as mean value and standard error of the mean (SEM). Statistical significance was determined using the Student's t-test, with p ≤ 0.05 being considered statistically significant.

## 3 Results

### 3.1 Biodistribution and dosimetry

**Fig. 1:**
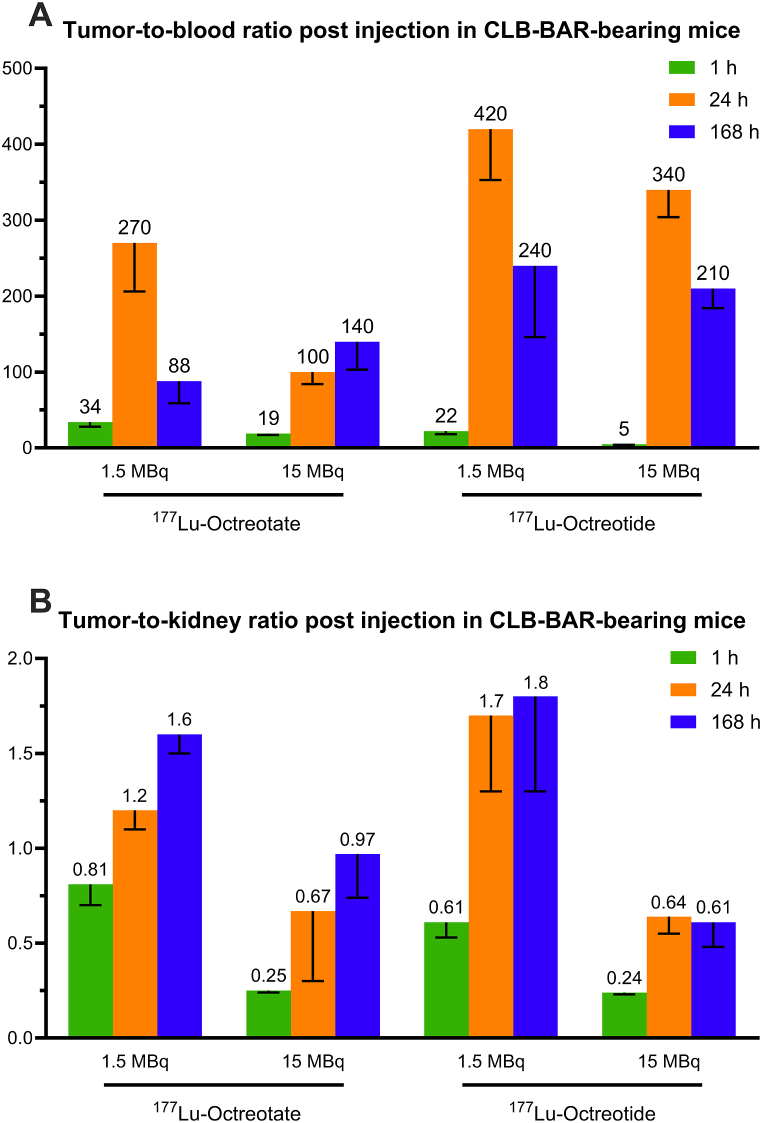
(**A**) Tumor-to-blood and (**B**) tumor-to-kidney ^177^Lu activity concentration ratios for CLB-BAR bearing mice, 1h, 24h and 168h post injection with ^177^Lu-octreotate (data from [27]) or ^177^Lu-octreotide (1.5 or 15 MBq). Error bars represent the standard error of the mean (SEM).

Results from our previous biodistribution study on ^177^Lu-octreotate [27] and the current ^177^Lu-octreotide biodistribution in CLB-BAR-bearing nude mice are presented in Table 1, Table 2. Data is given as ^177^Lu activity concentration per injected activity (%IA/g) in each tissue, together with tumor-to-normal-tissue activity concentration ratios (T/Ns) at 1, 24, and 168 h post-injection with 1.5 MBq or 15 MBq ^177^Lu-octreotate or ^177^Lu-octreotide (n = 5–6/group). The uptake in tumor tissue was higher for ^177^Lu-octreotate than ^177^Lu-octreotide, except for at 24 h post-injection with 15 MBq. The uptake of ^177^Lu-octreotate was also higher in most of the other organs. The highest ^177^Lu concentrations in the normal tissues were found in kidneys for both radiopharmaceuticals, with higher values for ^177^Lu-octreotate. The highest T/Blood and T/Kidney values were, however, observed for ^177^Lu-octreotide (Fig. 1 [A-B]). The mean absorbed dose to each tissue and tumor is presented in Table 3. The absorbed doses of ^177^Lu-octreotate were higher than those of ^177^Lu-octreotide in all tissues except the liver after administration of 15 MBq. The mean absorbed dose to the tumor was estimated to 4.4 Gy and 6.7 Gy after administration of 1.5 and 15 MBq ^177^Lu-octreotate, respectively. Corresponding values for ^177^Lu-octreotide were 1.1 Gy and 4.5 Gy.

**Table 1:** Biodistribution of 1.5 MBq^177^Lu-octreotate and 1.5 MBq^177^Lu-octreotide in CLB-BAR-bearing nude mice. Data is given as^177^Lu activity concentration (%IA/g) in each tissue and tumor-to-normal-tissue^177^Lu activity concentration ratios (T/N) at 1, 24 and 168 h after injection of 1.5 MBq^177^Lu-octreotate (data from Ref. [27]) or^177^Lu-octreotide (n = 5–6/group). Values are given as mean (SEM).

	1.5 MBq (^177^Lu-octreotate) – Reference [27]			1.5 MBq (^177^Lu-octreotide) – Present study		
^**177**^**Lu activity concentration (%IA/g)**						
**Time p.i.**	**1 h**	**24 h**	**168 h**	**1 h**	**24 h**	**168 h**
Adrenals	6.6 (1.6)	3.4 (0.5)	1.0 (0.2)	0.71 (0.10)	0.32 (0.10)	0.17 (0.01)
Blood	1.5 (0.13)	0.13 (0.04)	0.095 (0.05)	0.54 (0.06)	0.040 (0.007)	0.010 (0.002)
Bone marrow	–	–	–	4.9 (2.8)	1.7 (0.5)	0.43 (0.06)
Brain	0.28 (0.10)	0.074 (0.01)	0.027 (0.006)	0.069 (0.013)	0.025 (0.003)	0.013 (0.002)
Kidneys	61 (3)	22 (1)	2.8 (0.2)	18 (2)	10 (1)	0.95 (0.07)
Liver	2.0 (0.4)	0.52 (0.03)	0.22 (0.02)	0.55 (0.04)	0.37 (0.03)	0.14 (0.01)
Lungs	16 (2)	4.1 (0.9)	1.1 (0.1)	2.5 (0.1)	1.6 (0.2)	0.29 (0.03)
Spleen	5.1 (2.1)	1.1 (0.1)	0.65 (0.04)	0.57 (0.07)	0.29 (0.04)	0.16 (0.03)
Tumor	49 (8)	25 (1)	4.6 (0.6)	11 (1)	17 (4)	1.7 (0.3)
	**Tumor-to-normal-tissue concentration ratio (T/N)**					
**Time p.i.**	**1 h**	**24 h**	**168 h**	**1 h**	**24 h**	**168 h**
Adrenals	9.0 (2.0)	7.8 (0.7)	6.4 (1.9)	16 (2)	67 (21)	11 (5)
Blood	34 (6)	270 (64)	88 (29)	22 (4)	420 (67)	240 (94)
Bone marrow	–	–	–	4.0 (0.9)	14 (6)	4.3 (1.6)
Brain	240 (60)	360 (38)	200 (40)	170 (16)	700 (180)	140 (43)
Kidneys	0.81 (0.11)	1.2 (0.1)	1.6 (0.1)	0.61 (0.08)	1.7 (0.4)	1.8 (0.5)
Liver	28 (5)	50 (3)	21 (3)	20 (3)	46 (12)	12 (3)
Lungs	3.0 (0.4)	13 (7)	4.2 (0.6)	4.4 (0.6)	11 (2)	6.2 (2.3)
Spleen	14 (3)	25 (3)	7.3 (1.1)	20 (3)	58 (11)	10 (2)

**Table 2:** Biodistribution of 15 MBq^177^Lu-octreotate and 15 MBq Lu-octreotide in CLB-BAR-bearing nude mice. Data is given as^177^Lu activity concentration (%IA/g) in each tissue and tumor-to-normal-tissue^177^Lu activity concentration ratios (T/N) at 1, 24 and 168 h after injection of 15 MBq^177^Lu-octreotate (data from Ref. [27]) or^177^Lu-octreotide (n = 5–6/group). Values are given as mean (SEM).

	15 MBq (^177^Lu-octreotate) - Reference [27]			15 MBq (^177^Lu-octreotide) – Present study		
^177^Lu activity concentration (%IA/g)						
**Time p.i.**	**1 h**	**24 h**	**168 h**	**1 h**	**24 h**	**168 h**
Adrenals	1.8 (0.1)	0.16 (0.05)	0.26 (0.07)	1.6 (0.9)	0.18 (0.03)	0.055 (0.013)
Blood	0.78 (0.11)	0.0053 (0.0015)	0.009 (0.002)	0.93 (0.27)	0.014 (0.001)	0.0038 (0.0004)
Bone marrow	–	–	–	1.2 (0.1)	0.20 (0.04)	0.40 (0.16)
Brain	0.22 (0.05)	0.0078 (0.0008)	0.014 (0.002)	0.084 (0.011)	0.029 (0.004)	0.012 (0.000)
Kidneys	57 (6)	2.0 (0.7)	1.2 (0.1)	16 (1)	7.5 (0.3)	1.5 (0.3)
Liver	1.4 (0.2)	0.028 (0.008)	0.068 (0.003)	0.77 (0.09)	0.15 (0.01)	0.12 (0.01)
Lungs	3.8 (0.4)	0.14 (0.03)	0.14 (0.01)	1.6 (0.1)	0.21 (0.02)	0.065 (0.005)
Spleen	1.1 (0.2)	0.052 (0.010)	0.13 (0.01)	0.61 (0.05)	0.10 (0.01)	0.073 (0.008)
Tumor	14 (1)	0.53 (0.16)	1.5 (0.3)	4.0 (0.3)	4.6 (0.4)	0.82 (0.17)
	**Tumor-to-normal-tissue concentration ratio (T/N)**					
**Time p.i.**	**1 h**	**24 h**	**168 h**	**1 h**	**24 h**	**168 h**
Adrenals	7.7 (0.7)	3.3 (0.2)	5.1 (1.5)	5.1 (1.2)	27 (3)	25 (13)
Blood	19 (2)	100 (16)	140 (37)	5.0 (0.8)	340 (36)	210 (26)
Bone marrow	–	–	–	3.4 (0.4)	26 (6)	2.6 (0.6)
Brain	71 (14)	65 (15)	73 (13)	49 (6)	180 (39)	67 (13)
Kidneys	0.25 (0.01)	0.67 (0.37)	0.97 (0.23)	0.24 (0.01)	0.64 (0.09)	0.61 (0.13)
Liver	10 (1)	22 (4)	15 (3)	5.3 (0.5)	30 (3)	7.1 (1.7)
Lungs	3.7 (0.4)	3.7 (0.8)	6.4 (2.3)	2.4 (0.2)	22 (2)	13 (3)
Spleen	14 (2)	9.5 (1.6)	9.5 (3.0)	6.4 (0.6)	47 (6)	12 (2)

**Table 3:** Mean absorbed dose per amount of injected activity (Gy/MBq) to various organs in mice bearing CLB-BAR after injection with^177^Lu-octreotate (data from Ref. [27]) or^177^Lu-octreotide (present study). Data were corrected for decay before estimation of the absorbed dose. Data are given as mean (min, max), where the range was calculated based on data from Table 1, Table 2

Cell line Peptide	CLB-BAR ^177^Lu-octreotate (Reference [27])		CLB-BAR ^177^Lu-octreotide (present study)	
**Injected act.**	**1.5 MBq**	**15 MBq**	**1.5 MBq**	**15 MBq**
Adrenals	0.30 (0.24, 0.36)	0.096 (0.076, 0.11)	0.038 (0.033, 0.043)	0.033 (0.020, 0.042)
Blood	0.029 (0.021, 0.037)	0.0061 (0.0046, 0.0071)	0.0073 (0.0060, 0.0084)	0.0058 (0.0049, 0.0066)
Bone marrow	–	–	0.17 (0.11, 0.22)	0.081 (0.052, 0.11)
Brain	0.0091 (0.0070, 0.011)	0.0065 (0.0060, 0.0073)	0.0034 (0.0028, 0.0036)	0.0040 (0.0034, 0.0046)
Kidneys	2.7 (2.3, 3.1)	1.3 (1.1, 1.5)	0.65 (0.58, 0.72)	0.72 (0.64, 0.80)
Liver	0.067 (0.060, 0.072)	0.029 (0.025, 0.032)	0.036 (0.033, 0.039)	0.033 (0.030, 0.035)
Lung	0.46 (0.39, 0.53)	0.10 (0.087, 0.11)	0.12 (0.11, 0.13)	0.036 (0.022, 0.039)
Spleen	0.16 (0.14, 0.20)	0.049 (0.046, 0.055)	0.035 (0.030, 0.040)	0.021 (0.019, 0.022)
Tumor	2.9 (2.4, 3.4)	0.45 (0.37, 0.54)	0.74 (0.53, 0.96)	0.30 (0.24, 0.35)

### 3.2 Therapy

**Fig. 2:**
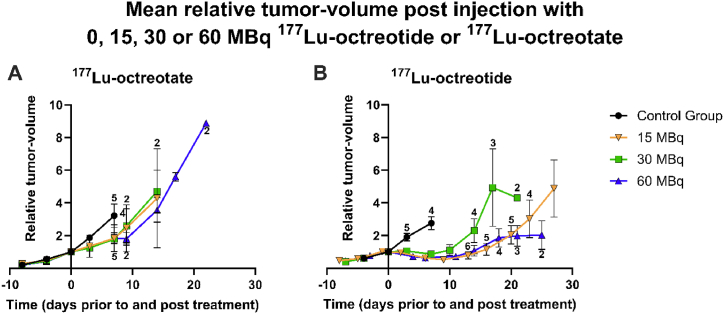
Mean relative tumor-volume post treatment with (**A**) ^177^Lu-octreotate or (**B**) ^177^Lu-octreotide (n = 4–6/group). The mice in the control group were injected i.v. with saline. The error bars represent the standard error of the mean (SEM) and the sample size is presented above or beneath the error bars. * represents statistically significant difference between the data for the control group (at day 7) and data for each of the treated groups (at day 6–7), respectively (p < 0.05).

The relative tumor volume was determined over time after single-administration of 15 MBq, 30 MBq, and 60 MBq of ^177^Lu-octreotate and ^177^Lu-octreotide (Fig. 2 [A-B]). For ^177^Lu-octreotate, no statistically significant differences were observed in relative tumor volume between the different treated groups and the control group. However, for ^177^Lu-octreotide, a clear difference was found between the control group and the different treatment groups. Regardless of the ^177^Lu-octreotide activity, all groups demonstrated a statistically significant difference in relative tumor volume compared to the control group after a week. No clear dose-response relationship was observed neither for ^177^Lu-octreotide nor for ^177^Lu-octreotate.

**Fig. 3:**
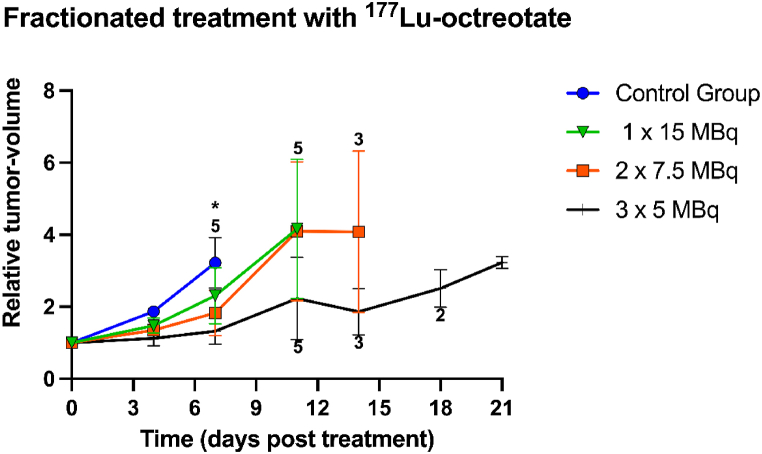
Mean relative tumor-volume after fractionated treatment with ^177^Lu-octreotate, totally 15 MBq per mouse. The mice were treated with 1 × 15 MBq, 2 × 7.5 MBq, or 3 × 5 MBq ^177^Lu-octreotate, with 2 h between the injections for group 2x7.5 and 1 h for group 3x5. The error bars represent the standard error of the mean (SEM) and the group size is presented above or beneath the error bars. * represents significant difference (p < 0.05) between the control group and 3 × 5 MBq group.

In general, fractionated administration seemed to result in more prominent tumor volume reduction than single administration (Fig. 3). On day seven, a statistically significant difference was observed in relative tumor volume between the control and the 3 × 5 MBq groups.

**Fig. 4:**
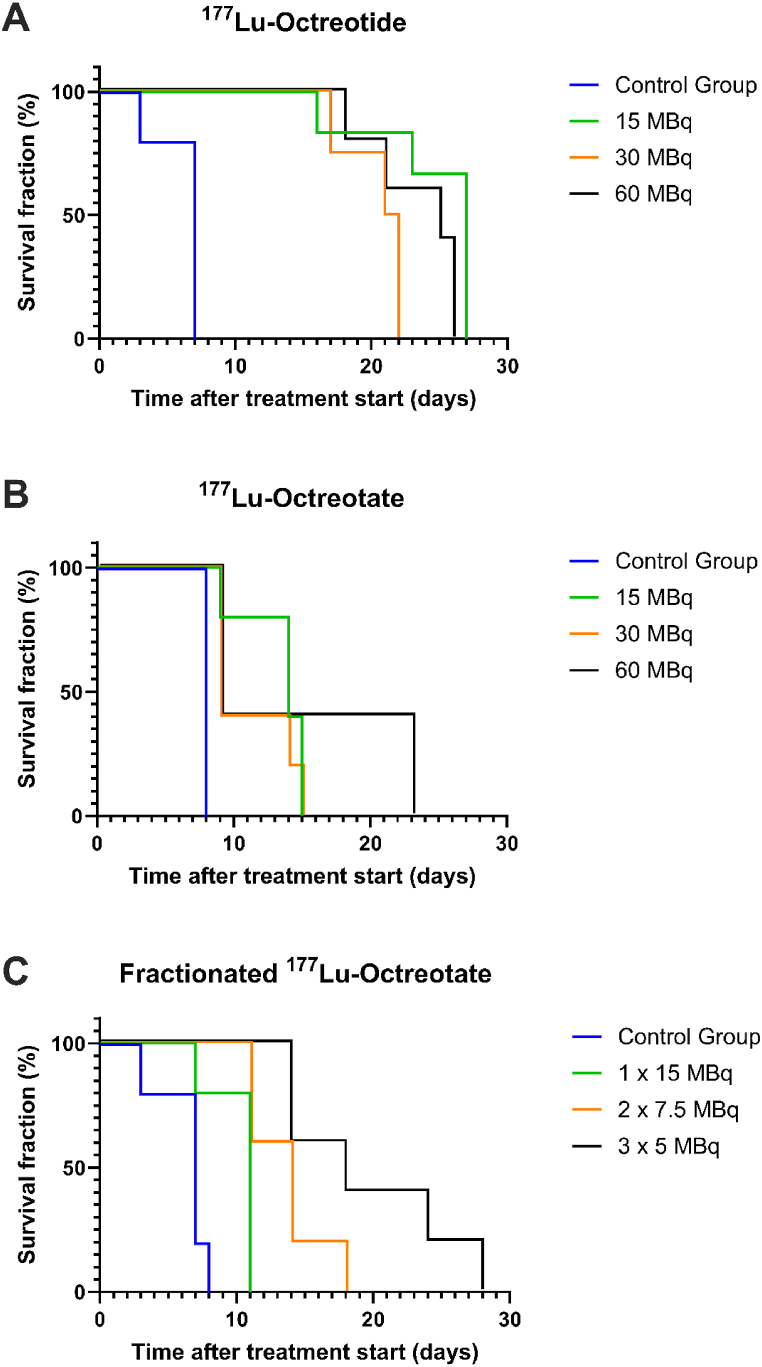
Kaplan-Meier analyses illustrating the overall survival fraction after treatment start. The mice were injected with various amounts of (**A**) ^177^Lu-octreotide, (**B**) ^177^Lu-octreotate, or (**C**) 1 × 15 MBq, 2 × 7.5 MBq, or 3 × 5 MBq ^177^Lu-octreotate, with 2 h between the injections for group 2 × 7.5 MBq and 1 h for group 3 × 5 MBq. The control groups were i.v. administered with saline. The animals were killed when the tumor mass exceeded 10 % of the body weight.

Kaplan-Meier analyses for overall survival (OS), where mice were sacrificed when tumor mass exceeded 10 % of body weight, demonstrated that treatment with single injected ^177^Lu-octreotide resulted in much longer median OS than controls (21–27 d *versus* 7 d) (Fig. 4 [A-C]). For ^177^Lu-octreotate given as single injection the median OS was somewhat better than for controls (9–14 d *versus* 8 d). Fractionated therapy with ^177^Lu-octreotate gave a better response than single administration. The median OS was 18 d after treatment with 3 × 5 MBq, and 14 d, 11 d and 7 d after 2× 7.5 MBq, 1× 15 MBq and saline (controls), respectively. Corresponding maximum OS values were 28 d, 18 d, 11 d and 8 d.

### 3.3 Regulation of apoptosis-related genes

In order to better characterize the tumor response we analyzed RNA from tumors collected when the animals were killed, 8–28 days after single-injection with ^177^Lu-octreotate or ^177^Lu-octreotide, or 2–8 days after saline injection for controls (n = 4–6/group).

**Fig. 5:**
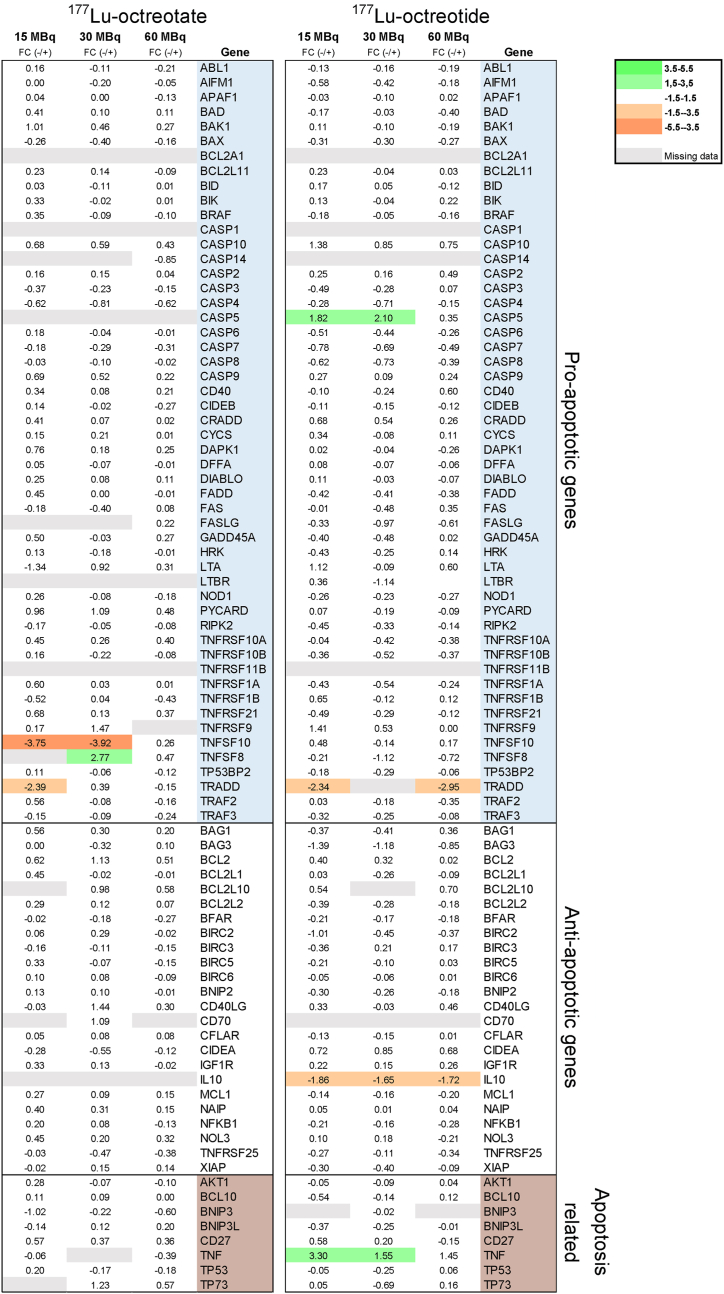
mRNA expression of pro-, anti-apoptotic genes and apoptosis related genes in tumor tissue (CLB-BAR) of mice injected with 15, 30 or 60 MBq ^177^Lu-octreotate (to the left) or ^177^Lu-octreotide (to the right) (n = 4–5/group). Gene regulation is expressed as fold change relative to the control group. Red and green represent down- and up-regulation, respectively, with │FC│>1.5. Missing data are represented by gray color. (For interpretation of the references to color in this figure legend, the reader is referred to the Web version of this article.)

**Fig. 6:**
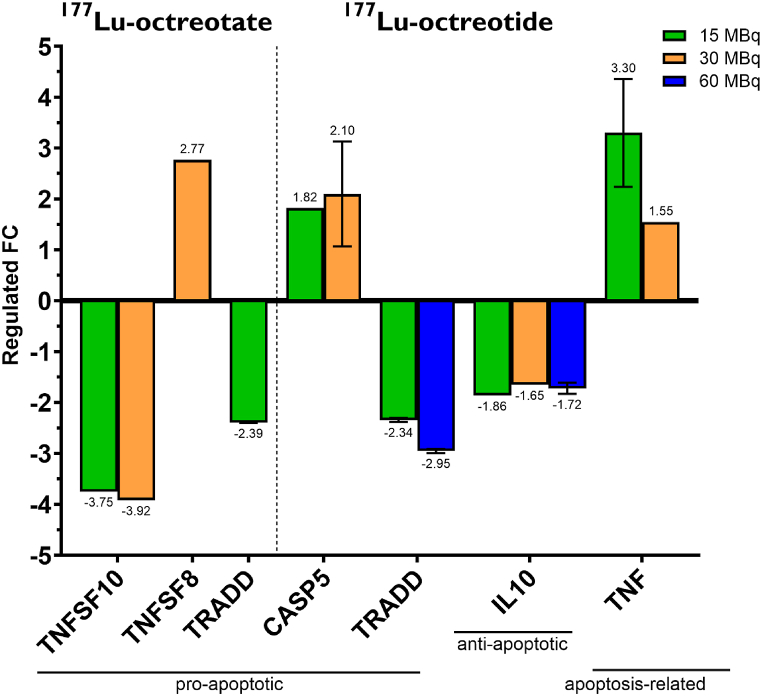
mRNA expression of the regulated genes in CLB-BAR tumors from mice after treatment with 15, 30 or 60 MBq ^177^Lu-octreotate (to the left of the dashed line) or ^177^Lu-octreotide (n = 4–6/group). The genes are categorized as pro-, and anti-apoptotic genes and apoptosis-related genes. Data are expressed as fold change (FC) relative the control group, with │FC│>1.5. The error bars represent the standard error of the mean (SEM).

After treatment with ^177^Lu-octreotate, expression of the pro-apoptotic genes *TNFSF8* (n = 1), *TNFSF10* (n = 1)*,* and *TRADD* (n = 2, p = 0.030) were differentially regulated (│FC│≥1.5) compared to controls (Fig. 5, Fig. 6). In the 15 MBq group, both *TNFSF10* and *TRADD* were down-regulated, while for the 30 MBq group, *TNFSF10* was down-regulated, and *TNFSF8* showed an up-regulation. On the contrary, no genes were differently regulated after treatment with 60 MBq ^177^Lu-octreotate. Also, neither anti-apoptotic, nor “apoptosis-related” genes, classified as both pro-and anti-apoptotic, were differentially regulated compared to controls.

Administration of ^177^Lu-octreotide resulted in differential expression of *CASP5* (15 MBq, n = 1, and 30 MBq, n = 2, p = 0.091), *IL10* (15 MBq, n = 1, 30 MBq, n = 1, and 60 MBq, n = 2, p = 0.045)*, TNF* (15 MBq, n = 2, p = 0.054, and 30 MBq, n = 1)*,* and *TRADD* (15 MBq, n = 2, p = 0.008, and 60 MBq, n = 2, p = 0.010) (Fig. 5, Fig. 6). *CASP5, IL10*, and *TNF* are classified as pro-apoptotic, anti-apoptotic, and apoptosis-related genes, respectively. In both 15 MBq and 30 MBq groups, the *CASP5* and *TNF* genes were differentially up-regulated. In all treated groups the *IL10* gene was s differentially downregulated, whereas only tumors in the 15 MBq and 60 MBq groups showed significant downregulation of the *TRADD* gene.

Comparing the effects of treatment with ^177^Lu-octreotate and ^177^Lu-octreotide, *TRADD* was commonly down-regulated in both 15 MBq group. No other commonly differentially regulated genes were identified.

## 4 Discussion

Significant advances have been made in treating patients with NB in the past few decades. Although the 10-year overall survival rate for NB patients has increased to approximately 80 %, the overall survival rate for high-risk NB patients is still low [8]. Novel treatment options are required for patients with high-risk NBs, where radiolabeled SSTAs may be an option if certain conditions are met (e.g., high expression of SSTRs and high radiosensitivity). In the present study, we examined how different activity levels of ^177^Lu-octreotate or ^177^Lu-octreotide influenced the biodistribution and therapeutic effects in a human high-risk NB mouse model. Furthermore, radiation-induced effects on expression of genes involved in apoptosis were studied.

According to the biodistribution data, ^177^Lu concentration in tissues was generally higher after administration of ^177^Lu-octreotate than ^177^Lu-octreotide, which resulted in higher absorbed dose to almost all tissues. It is also essential to take into consideration the relationship between the uptake in tumor and the dose-limiting organs, primarily kidneys and bone marrow. At all-time points after administration of 1.5 MBq ^177^Lu, higher T/Kidney and T/Blood values were observed for ^177^Lu-octreotide than ^177^Lu-octreotate. However, this trend was not demonstrated for 15 MBq, where T/Kidney values were higher for ^177^Lu-octreotate at all time-points. Bone marrow collected after treatment with ^177^Lu-octreotide, had the third highest mean absorbed dose after administration with 1.5 MBq and 15 MBq. T/Bone-marrow values were the highest 24h post-injection, with values of 14 and 26 for 1.5 MBq and 15 MBq, respectively. The reasons for differences in tumor uptake, as well as uptake and retention in normal tissues, is most probably differences in SSTR subtype expression between tissues, and differences in binding affinity to and internalization of the SSTR subtypes between the two SSTAs. Altogether, it is also clear that the obtained T/Ns are high in comparison with previous biodistribution data for other human NET animal models, such as GEP-NETs [[38], [39], [40], [41], [42], [43]], indicating the possibility of successful PRRT in patients with high-risk NB.

In the present study in human NB-bearing mice the uptake in kidneys was much higher at earliest time-points compared to biodistribution data from other human NET animal models, such as GEP-NETs [[38], [39], [40], [41], [42], [43]]. No kidney protecting agent was used in any of these studies. Possible explanations for the higher kidney uptake in the present study for both radiopharmaceuticals are: 1) different supplier of the nude mice, 2) different age of the mice, where the mice in the present study were adult but younger than those used for our GEP-NET studies, and 3) differences in influence by hormones produced by the different neuroendocrine tumor types.

When comparing the biodistribution data between the radiopharmaceuticals, the difference in specific activity, 25 MBq/μg for ^177^Lu-octreotate and 66 MBq/μg for ^177^Lu-octreotide, has most probably influenced the results. For ^177^Lu-octreotide, about 2.6 times fewer peptide molecules were administered for each activity level compared to ^177^Lu-octreotate. High specific activity is preferred, since a higher proportion of SSTA molecules being labeled with ^177^Lu, will lead to less saturation of the SSTRs at the cell surface (and hence higher binding and uptake) [44]. Despite this fact, the uptake in tumor tissue was highest for ^177^Lu-octreotate. However, the saturation effects were higher for octreotate since a 10-fold increase in activity level only led to a 1.5-fold increased mean absorbed dose to the tumor, compared with a 4-fold increase for octreotide.

After treatment with ^177^Lu-octreotide, an evident tumor volume reduction was observed compared to the control group for all activity levels studied. However, a clear dose-response relationship was not observed, supporting the suggestion of SSTR saturation effects. Treatment with ^177^Lu-octreotate resulted in a more modest tumor reduction. Saturation may also partly explain the lack of a dose-response relationship in tumor volume reduction for ^177^Lu-octreotate. For practical reasons, some groups receiving ^177^Lu-octreotide were measured on different days after treatment than the majority of the groups. This is unfortunate since it makes firm statistical comparisons difficult, but the overall appearance of the curves indicates a tumor volume reduction followed a later regrowth, which was not seen in the control groups or ^177^Lu-octreotate groups.

Based on the dosimetric estimations, the absorbed dose to the tumor from 15 MBq injected activity should be comparable or higher for ^177^Lu-octreotate than ^177^Lu-octreotide, thus leading to a comparable or more significant tumor volume reduction. However, ^177^Lu-octreotide demonstrated a more evident anti-tumor effect at this activity level, which was not expected and cannot be explained with certainty. One potential explanation could be that mice administered with ^177^Lu-octreotate had tumor volumes with a somewhat greater dispersion at the start of the treatment than those administered with ^177^Lu-octreotide. This could have contributed to higher variations within the ^177^Lu-octreotate groups, following treatment. These experiments were restricted by the logistics of ^177^Lu delivery and the timing of appropriate tumor sizes in the animals. However, the tumor growth rate before administration was similar between groups, and based on our long experience of these types of studies, this explanation is not plausible. The biodistribution data for ^177^Lu-octreotate was also taken from a previous study using the same xenograft model [27], and possible batch effects cannot be ruled out. Additional studies are needed to fully explain this phenomenon.

The discussion above regarding the differences in therapeutic effects was based on radiobiological effects only, which in most similar studies can explain the results. However, if the SSTAs by themselves would influence the therapeutic effect, then lower specific activity (with kept activity level) means that more peptide molecules are administered. This would then gain therapeutic effects from ^177^Lu-octreotate compared with ^177^Lu-octreotide, if we assume that the two SSTAs would give similar effects. However, the binding profile to the five SSTR subtypes differ between the SSTAs, and a direct comparison is not possible without data on the therapeutic effect of unlabeled octreotate and octreotide on NBs.

In previous comparative *in vivo* studies with radiolabeled octreotate or octreotide, the former yielded higher activity concentrations in the tumor and more significant anti-tumor effects [29,30]. The animal models in these studies were nude mice bearing human small-intestine NET (GOT1) or human small-cell lung cancer (NCI–H69) tumors. In these studies, the specific activity was similar within each study, while in the NCI–H69 study the SSTAs were labeled differently (^111^In-DTPA-octreotide *vs.* ^177^Lu-DOTA-octreotate), which would also influence the biodistribution [29,30]. When comparing ^111^In-DOTA-octreotide and ^111^In-DOTA-octreotate in patients with NETs, radiolabeled octreotate had the highest uptake in most tissues, but octreotide was found to be slightly more beneficial, exhibiting higher T/Kidney values in some patients [45]. Similarly, in another clinical study, patients with NETs were treated with ^177^Lu-octreotide or ^177^Lu-octreotate, where the latter was considered more beneficial in terms of higher absorbed dose delivered to the tumor [46].

The fractionation study with ^177^Lu-octreotate showed a more prominent response in the group treated with 3 × 5 MBq (a hyper-fractionated scheme), similar to what has previously been demonstrated in the GOT1 GEP-NET mouse model [47]. SSTR2 internalization can occur within a few minutes and be recycled within 40 min, an advantage which might allow an administration interval of 1 h to be efficient [48,49]. The kinetics of internalization and recycling might however differ for different SSTR subtypes and tumor types. To optimize uptake in the tumor in relation to the normal tissue, the effects of fractionation and receptor internalization, recycling, and affinity for SSTAs need to be studied in more detail.

HR-NBs are characterized by their therapy-resistant features, with the oncogenes *MYCN* and *p53* playing a prominent role in this process. The *MYCN* gene plays a critical role in impairing apoptosis and stimulating tumor growth, contributing to reduction of therapeutic effects [[50], [51], [52]]. Mutations in *p53* are not frequently detected in *MYCN*-amplified NBs at time of diagnosis, but mutations are often acquired after treatment, leading to therapy resistance in relapsed HR-NBs [53]. Although prior studies have promoted apoptosis and increased differentiation in HR-NB by silencing MYCN, selectivity remains an issue [50].

To optimize treatment with ^177^Lu-octreotate or ^177^Lu-octreotide, it is of great importance to evaluate tumor response, not only by analyzing the tumor volume, but also by exploring radiobiological effects in more detail. In the present study, we focused on 84 genes involved in apoptosis, since it is well established that ionizing radiation induces apoptosis. In total, only six of these genes were regulated differentially (│FC│>1.5), despite the relatively high absorbed dose delivered to the tumors. This finding clearly demonstrates the therapy-resistant nature of NBs, which also was confirmed by the small reduction in tumor volume and lack of dose-response relationship.

The three genes that were found differently regulated after treatment with ^177^Lu-octreotate were all classified as pro-apoptotic, and commonly known as members of the tumor necrosis factor (TNF) superfamily (TNFSF) or TNF receptor superfamily (TNFRSF). *TNFSF10* (TNFSF member 10) was down-regulated and this gene encodes for a membrane-bound cytokine (TNFSF10/TRAIL/Apo2L), which initiates apoptosis by binding to death receptor (DR) 4 and/or DR5 [54,55]. *TRADD* (TNFRSF1A-associated via death domain) was also down-regulated and encodes for TRADD, an adaptor protein involved in the recruitment and activation of caspases, following the binding of TNF to TNFR1, which can lead to apoptosis [56]. Moreover, it was previously reported that DR6-mediated apoptosis can also be initiated by TRADD [57,58]. Interestingly, *TNFSF8* was up-regulated in the 15 MBq group, and encodes for an additional cytokine that binds to TNFRSF8 (CD30). Activation of CD30 can induce apoptosis and hinder the activation of the transcription factor NF-κB, which is known to be involved in crucial pro-survival processes [59]. NF-κB itself is also involved in pro-inflammatory response indicating its target potential for various diseases [60]. Altogether, these findings support the weak therapeutic effect on HR-NB tumor volume that was observed for ^177^Lu-octreotate.

Treatment with ^177^Lu-octreotide yielded differential regulation of 4 genes. The pro-apoptotic *CASP5* (*Caspase 5*) was up-regulated (15 MBq/30 MBq) and *TRADD* down-regulated (15 MBq/60 MBq). CASP5 is an inflammatory caspase and involved in pyroptosis [61]. The anti-apoptotic gene, *IL-10* (*Interleukin 10*), encoding a cytokine with anti-inflammatory properties, was down-regulated in all groups treated with ^177^Lu-octreotide [62]. This may indicate a radiation-induced inflammatory response. Lastly, *TNF* was found up-regulated (15 MBq/30 MBq) and classified as an apoptosis-related gene, since TNF is involved in both tumor-suppressing and -promoting signaling pathways, depending on its binding to TNFR1 or TNFR2, respectively [63]. The binding of TNF to TNFR1 leads to apoptosis via the same pathway as TRADD, and the binding of TNF to TNFR2 promotes the pro-survival of NF-κB pathway [63,64].

It is known that ionizing radiation can activate both the intrinsic and the extrinsic apoptotic pathways [65,66]. In the present study we mainly found effects related to the extrinsic pathway. Previous investigations have illustrated how the global gene expression changes over time after exposure, and that the apoptosis-related genes were mainly regulated three to six days post-treatment [[67], [68], [69]]. We previously demonstrated how the apoptotic cell count reached its maximum one and three days after injection with ^177^Lu-octreotate in nude mice with human GOT1-tumors, accompanied by a pronounced reduction on day seven [70]. However, in the present study we intended to analyze the change in tumor volume over a more extended period. Thus, this prioritization restricted our findings regarding early-responding genes involved in apoptosis. The present findings still demonstrate a higher impact on the activation of the extrinsic apoptosis pathway for ^177^Lu-octreotide compared to ^177^Lu-octreotate. This indicates a possible mechanism for higher therapeutic response. Future studies should also include earlier time points for a better understanding.

With the drug-resistant properties of HR-NB, a multimodal form of treatment will most probably be preferable. Furthermore, considering the advantage that each treatment may act on different organs at risk, the anti-tumor effects can be optimized. Our study shows that radiolabeled SSTAs can be beneficial in disseminated SSTR-positive HR-NB. The tumor-seeking properties of radiolabeled SSTAs could be utilized in combination with radiosensitizing drugs to enhance its anti-tumor effects. For example, a previous study has reported favorable outcomes of combining ^177^Lu-octreotate with an MDM2/4 inhibitor in mouse models of neuroblastoma [71]. It would e.g. be of interest to examine PRRT combined with ALK TKI, in order to study how inhibition of the ALK signaling pathway affects the anti-tumor effects following radiation.

## 5 Conclusion

The choice of ^177^Lu-labeled SSTA affected the biodistribution and anti-tumor effects in mice bearing the human HR-NB CLB-BAR. The therapeutic effects (of both analogs) were modest compared with previous studies in GEP-NET mouse models. The mice that received single administration of ^177^Lu-octreotide displayed a more substantial anti-tumor effect than after single administration of ^177^Lu-octreotate, despite the potentially higher mean absorbed doses to tumors from ^177^Lu-octreotate. Future studies should investigate the effects of combining ^177^Lu-labeled SSTAs with radiosensitizing agents.

## Ethics statement

All animal experiments were approved by the Swedish Ethical Committee on Animal Experiments in Gothenburg (ethical reference numbers: 107-15 and 2779-20) and carried out following guidelines from Animal Research: Reporting of In Vivo Experiments (ARRIVE)

## Funding

This study was supported by grants from the 10.13039/501100004359Swedish Research Council (EFA: 2021–02636; RHP: 2019–03914; BH: 2021-01192), the 10.13039/501100002794Swedish Cancer Society (EFA: CAN20/1293, 23 2975; RHP: CAN21/1459; BH: CAN21/1525), 10.13039/501100006313Swedish Childhood Cancer Foundation (EFA: PR2017-0057; RHP: PR2022-0029; BH: PR2021-0027), BioCARE - a National Strategic Research Program at 10.13039/501100005760University of Gothenburg, the Swedish state under the agreement between the Swedish government and the county councils – the ALF-agreement (ALFGBG-966074), the King Gustav V Jubilee Clinic 10.13039/100002002Cancer Research Foundation, the 10.13039/501100005754Sahlgrenska University Hospital Research Funds, Wilhelm and Martina Lundgren Research Foundation, Assar Gabrielsson 10.13039/100002002Cancer Research Foundation, Herbert & Karin Jacobsson Foundation, and 10.13039/501100014552Adlerbertska Research Foundation.

## Data availability

All data is available in the manuscript and references.

## CRediT authorship contribution statement

**A. Romiani:** Writing – review & editing, Writing – original draft, Visualization, Validation, Investigation, Funding acquisition, Data curation, Conceptualization. **K. Simonsson:** Writing – review & editing, Validation, Investigation. **D. Pettersson:** Writing – review & editing, Investigation. **A. Al-Awar:** Writing – review & editing, Investigation, Funding acquisition. **N. Rassol:** Writing – review & editing, Investigation. **H. Bakr:** Writing – review & editing, Investigation, Funding acquisition. **D.E. Lind:** Writing – review & editing, Investigation. **G. Umapathy:** Writing – review & editing, Investigation. **J. Spetz:** Writing – review & editing, Visualization, Methodology. **R.H. Palmer:** Writing – review & editing, Resources, Funding acquisition, Conceptualization. **B. Hallberg:** Writing – review & editing, Resources, Funding acquisition, Conceptualization. **K. Helou:** Writing – review & editing, Supervision, Methodology, Conceptualization. **E. Forssell-Aronsson:** Writing – review & editing, Visualization, Supervision, Resources, Project administration, Methodology, Funding acquisition, Data curation, Conceptualization.

## Declaration of competing interest

The authors declare that they have no known competing financial interests or personal relationships that could have appeared to influence the work reported in this paper.
